# Biological Assessment of Potential Exposure to Occupational Substances in Current Semiconductor Workers with at Least 5 Years of Employment

**DOI:** 10.3390/ijerph19148737

**Published:** 2022-07-18

**Authors:** Kyungsik Kim, Ho Kyung Sung, Jieun Jang, Chang-Mo Kang, Kwan Lee, Sue K. Park

**Affiliations:** 1 Department of Preventive Medicine, College of Medicine, Seoul National University, Daehak-ro 101, Jongno-gu, Seoul 03080, Korea; kks6235@snu.ac.kr (K.K.); yanghyo25@snu.ac.kr (J.J.); 2 Department of Biomedical Science, Graduate School, Seoul National University, Daehak-ro 101, Jongno-gu, Seoul 03080, Korea; 3 Cancer Research Institute, College of Medicine, Seoul National University, Daehak-ro 101, Jongno-gu, Seoul 03080, Korea; 4 National Medical Center, Institute for Public Healthcare, Eulji-ro 245, Jung-gu, Seoul 04564, Korea; hokyungsung@nmc.or.kr; 5 National Emergency Medical Center, National Medical Center, Eulji-ro 245, Jung-gu, Seoul 04564, Korea; 6 Gyeongnam Center for Infectious Disease Control and Prevention, Jungang-daero 300, Uichang-gu, Changwon 51154, Korea; 7 Research Institute of Radiological & Medical Sciences, Korea Institute of Radiological & Medical Sciences, Nowon-ro 75, Nowon-gu, Seoul 01812, Korea; kangcm@kirams.re.kr; 8 Department of Preventive Medicine, College of Medicine, Dongguk University, Gyeongju 38066, Korea; kwaniya@dongguk.ac.kr; 9 Integrated Major in Innovative Medical Science, College of Medicine, Seoul National University, Daehak-ro 101, Jongno-gu, Seoul 03080, Korea

**Keywords:** biological exposure indices, carcinogen, occupational substances, semiconductor worker, surrogate marker

## Abstract

Background: this study aimed to conduct a biological assessment of the potential exposure to carcinogenic substances in current semiconductor workers. Methods: A cross-sectional study was conducted on 306 semiconductor workers. The assessed biomarkers were as follows: (benzene) urine S-phenylmercapturic, trans,trans-muconic acid, blood benzene; (trichloroethylene) urine trichloroacetic acid; (2-ethoxyethanol) 2-ethoxyacetic acid; (arsine) urine arsenic3+, arsenic5+, monomethylarsonic, dimethylarsinic acid, arsenobetaine; (shift work) 6-hydroxymelatonin; (smoking) cotinine, and (radiation). The detection rate of these materials is defined as more than the biological exposure index (BEI) or the previous reference value. Results: Some workers exposed to trans,trans-muconic acid, trichloroacetic acid, and arsenic5+ showed high BEI levels. Generally, there was no difference according to job categories, and workers were suspected to be exposed to other sources. The melatonin concentration tended to decrease when working at night, and cotinine was identified as an excellent surrogate marker for smoking. In the case of radiation exposure, there was no significant difference in the number of stable chromosome translocation in 19 semiconductor workers. Their estimated radiation exposure level was below the limit of detection (LOD) or near the LOD level. Conclusion: In this study, most carcinogens were below the BEI level, but verification through re-measurement was needed for workers who were identified to have a high BEI level. For continuous monitoring, a prospective cohort is necessary to deal with the healthy worker effect and assess additional materials.

## 1. Introduction

Since the beginning of the semiconductor industry in the 1960s, it has continued to develop worldwide [1]. The global semiconductor market has been increasing, including the Asia-Pacific semiconductor market, especially in Taiwan and South Korea [2]. Therefore, it is expected to produce enormous economic effects and establish continuous employment and investment. However, since the 1980s, various diseases, including musculoskeletal disease, reproductive system disease, and cancer, have been reported in semiconductor workers [3,4,5,6]. This has raised hypotheses regarding the relationship between semiconductor work and occupational diseases.

The first epidemiological occupational study from the United Kingdom reported that the risk of cancer incidence and death were high in the semiconductor industries [6]. Since then, many studies have been conducted to assess the association between occupational exposure in the semiconductor industry and occupational diseases as a cohort or systematic review [7,8,9,10]. However, it was difficult to assess causality because of the controversy between the studies.

Leukemia was reported in a female semiconductor worker in her 20s in 2007 in South Korea. This raised social interest in semiconductor workers’ health and research on occupational exposure and diseases in semiconductor workers. As a result of this case, compensation measures for workers with similar problems and preventive measures for the health problems of semiconductor workers have emerged as major issues and an epidemiological investigation should be made about which hazardous occupational substances semiconductor workers may have been previously exposed to. Moreover, benzene showed rare exposure in the semiconductor environment, but a previous study reported that there was little exposure in a Korean semiconductor facility [11]. Therefore, this study aimed to assess occupational substances, such as benzene, in semiconductor workers. Along with this demand, we attempted to assess the level of previous and present occupational exposure to carcinogens. As a hypothesis, the concentrations of the biomarkers for occupational substances in the exposed group is higher than those in the non-exposed group, and long-term workers have been previously exposed to any occupational substances. Additionally, although the exposure level of workers to occupational substances is low in the current environment, they can be identified through biological assessment.

Occupational substance selection was based on the following three processes: (1) carcinogen and reproductive toxicity criteria from the Korea Occupational Safety and Health Agency (KOSHA) and International Agency for Research on Cancer (IARC) [12,13]; (2) expert opinion; and (3) additional reproductive toxicity materials or candidate exposure materials in semiconductor workers. Among the candidate materials, previous exposure to trichloroethylene (TCE) is suspected in the semiconductor assembly process. However, TCE has not been used by companies since 1995. As a byproduct, trichloroacetic acid (TCA) or trichloroethanol (TCOH) is excreted in urine and can be assessed during shift work and break times [14,15]. However, TCA was selected as an appropriate TCE biomarker because TCOH is excreted in the form of glucuronide conjugates or via exhalation [16]. Second, previous exposure to 2-ethoxyethanol (2-EE) is suspected in the photolithography process. As a byproduct, 2-ethoxyacetic acid has been identified in urine in both animal and human studies [17]. Furthermore, 2-EE is a hazardous reproductive substance that damages the testes and leads to infertility. The metabolism of 2-EE occurs in the bone marrow through the cytochrome P-450 system, which can be involved in leukemia [18]. During oxidation and metabolism, benzene is converted into S-phenylmercapturic acid and then excreted in the S-phenylglutathione form combined with glutathione [18,19]. Subsequently, trans,trans-muconic acid is excreted in urine as an additional metabolite of benzene [19,20]. Thus, we selected trans,trans-muconic acid, and to compensate for the low sensitivity and specificity, S-phenylmercapturic acid was included. Moreover, blood benzene was included as a benzene indicator, along with S-phenylmercapturic acid and trans,trans-muconic acid. Arsine (AsH3) is used in the ion implant process in the semiconductor industry, and ion implant workers can be exposed to arsenic, as arsine’s byproduct. Arsenic can be classified as arsenic (As3+), arsenic (As5+), monomethylarsonic acid (MMA), dimethylarsinic acid (DMA), and arsenobetaine (AsB) [21]. Arsenic exposure in the occupational environment is mainly inorganic arsenic exposure, which is more toxic than organic arsenic [22]. Moreover, arsenic (As)3+ is more toxic than As5+ [23]. Therefore, we conducted a speciation analysis to classify the arsenic species. Moreover, arsenic assessment in the blood is not suitable for semiconductors because it applies to chronic exposure (drinking water) or acute high-concentration exposure [24]. Additionally, to assess other sources, we considered the workers’ dietary information. Ionizing radiation is a known risk factor for cancer [13], and exposure to ionizing radiation is noted in the ion implant process in the semiconductor industry. Therefore, it is included as a candidate occupational substance in semiconductor work. Particularly, KOSHA published a study entitled “Guideline for Semiconductor Industry Workers” in 2012 [12]. It lists occupational substances in the semiconductor industry. Among the biomarkers described above and other materials that are carcinogenic and lead to reproductive toxicity as Group 1A from IARC, some materials are excluded for the following reasons: (1) ethanol for alcohol consumption; (2) measured by air monitoring (sulfuric acid, ethylene oxide, carbon monoxide, and nitrogen dioxide); (3) lung sediment (silicon); (4) materials exposed to various environments other than occupational exposure (formaldehyde); and (5) materials that are difficult to measure because of their short half-life (PGMEA, PGME) [24]. Furthermore, health effects were reported in shift workers [25]. Therefore, shift-work assessment is needed for semiconductor workers. Additionally, cotinine was assessed to evaluate smoking status [26,27]. The detailed assessment methods and rationales are described in Table S1.

This study aimed to evaluate the exposure level of the occupational substances (ionizing radiation, arsine, benzene, 2- ethoxyethanol, and trichloroethylene) which semiconductor workers were exposed to in the past or are likely to be exposed to. Furthermore, materials (cotinine, melatonin) that represent a semiconductor working environment were assessed in this study. In this study, we referred to both urine and blood samples to assess their exposure level based on the biological exposure index (BEI) in consideration of various semiconductor working environments.

## 2. Materials and Methods

### 2.1. Study Design and Population

This cross-sectional study assessed occupational substance levels based on BEI in current South Korean semiconductor workers. To select the representative study population, the type of work (semiconductor and subcontractor workers), work district (Giheung, 8-inch wafer size; Hwaseong, 12-inch wafer size; Asan, LCD; and Onyang, assembly), fab (line), job duties (office work, preventive maintenance, assembly package, ion implant process, non-implant processes, LCD, and operator), sex, and work duration (5–9 years, and >10 years) were considered (Figure S1). We identified eligible participants and selected the study population using stratified random sampling. First, we focused on the study population with work experience > 10 years and 5–9 years. Moreover, the shift work pattern was considered a 1:1:1 ratio as “day (06:00–14:00)”, “swing (14:00–22:00)”, and “night (22:00–06:00)”. As the study population, subcontractor workers who may be exposed to arsenic were selected as ion implant workers. As detailed, population information, LCD, non-implant, implant, and preventive maintenance workers consisted of men only, operators consisted of women only, and other groups consisted of both men and women. Considering the cumulative effect of exposure to occupational substances, current workers included those whose employment period is at least five years. Therefore, the range of the age was from 23 years to 50 years old, and they have been working between 1984 and 2012.

### 2.2. The Target Population of Each Occupational Exposure Substances

For each occupational exposure substance, semiconductor production workers collected their biospecimens twice (before and after work; Table S2). The office workers collected samples only once. In the case of benzene, we collected whole blood for blood benzene and urine samples for S-phenylmercapturic acid and trans,trans-muconic acid in semiconductor production workers. In the case of TCA and 2-ethoxyacetic acid, all workers assessed their concentrations in the urine samples (Table S2). To assess arsenic concentration, only subcontractor workers and the ion implant process workers were defined as the exposure group, and office and assembly package workers as an unexposed group were included. In the case of melatonin and cotinine, all workers collected their urine. To evaluate ionizing radiation exposure, ion implant process workers were included as the exposure group and office or assembly workers were included as the unexposed group. Their samples were all collected at once (after work).

### 2.3. Biological Exposure Indices

In this study, the BEI was used to assess the exposure status at the biological level (Table S1). This indicates that the highest mean concentration value (threshold limit value –time-weighted average) has no hazardous effect when workers are exposed to hazardous materials at the threshold limit value. In the USA, the American Conference of Governmental Industrial Hygienists (ACGIH) provided the standards, while KOSHA presented the Korean standards [28,29]. ACGIH recommended that BEI is a well-known indicator for biological monitoring in the occupational workers to assess the exposure status which does not adversely affect workers’ health based on the bio-specimen sample (blood, urine). In this regard, the BEI standard is relevant in assessing the health status of occupational substances on current semiconductor workers as a guidance value for biological monitoring. Moreover, by applying the BEI standard, we could compare the reference between ACGIH and KOSHA to define the reference indicator in each study; therefore, it is referred to in this study. When the BEI of occupational substance materials was compared between ACGIH and KOSHA, TCA, trans, trans-muconic acid, and S-phenylmercapturic acid were applied to ACGIH standards [28]. Blood benzene was applied to the KOSHA [29]. Others use both the ACGIH and KOSHA standards because they have the same BEI standards [28,29]. In the case of arsenic, the total hazardous level was estimated for both inorganic arsenic (As3+, As5+) and organic arsenic (DMA, MMA). For melatonin, we referred to a previous reference value because it was not possible to obtain BEI information [30]. In the case of blood benzene, 5 µg/L was applied according to the KOSHA standard, and 25 µg/gCr from ACGIH was applied to the S-phenylmercapturic acid standard. In the case of trans, trans-muconic acid, 500 µg/gCr, and 15 mg/L for TCA and 100 mg/gCr for 2-ethoxyacetic acid were applied based on the ACGIH standard. For arsenic, the total hazardous level was defined as 35 µg/L from ACGIH, and inorganic arsenic (As3+, As5+) was applied to the standard Agency for Toxic Substances and Disease Registry standard.

### 2.4. Samples and Information Collection

To collect the samples from the semiconductor workers, we considered that the working shift in 24 h has three rotation shifts (“day”, “swing”, “night”), semiconductor workers can be considered in two scenarios; (1) first working day after the holidays and (2) continuous semiconductor work (Figure S2). Therefore, we conducted sample collection and completed a self-questionnaire 1 h before work time and second samples (after work) were collected after working hours by double checking the questionnaire. To prevent sample deterioration, the collected samples were stored in a portable refrigerated storage box. Data from office workers were collected at once because of the low possibility of occupational exposure. According to the sample type, urine samples were collected in conical tubes, and blood samples were collected in three types: (1) whole blood, (2) plasma, and (3) serum. For the whole blood sample, benzene was assessed in ethylenediaminetetraacetic acid tubes, and radiation exposure was assessed in a heparin pretreatment tube. The detailed sample collection protocols and numbers for each job category are shown in Figure S2 and Table S3. Additionally, all the workers were investigated through the questionnaire in this study. A detailed questionnaire contains occupational history including employment, fabrication work status, shift work experience; individual disease history including cancer, drug, medical examination history; and lifestyle factors (smoking, drinking, and dietary consumption). Among the occupational history, their working department including semiconductor processes and job categories were referred from the human resource department.

### 2.5. Statistical Analysis

To assess the difference between the detection rate above the BEI level and job category in arsenic and melatonin, age-adjusted logistic regression was conducted. For status change according to the job category, a generalized estimating equation analysis was conducted to assess the statistical difference. Furthermore, the difference in geometric mean and proportion distribution according to the shift work pattern, duration, and bedtime was assessed by a generalized linear model and logistic regression, respectively. In the case of cotinine, statistical difference was assessed in each smoking status (non-smoker, former smoker, or current smoker) according to the job category. In this study, the kappa index was used as a consistency test for smoker definition between self-questionnaire and cotinine assessment through urine samples. The kappa index calculation aimed to assess the reliability of the cotinine assessment as smoker identification. The kappa index classification is as follows; 0.00–0.20, poor; 0.21–0.40, reasonable; 0.41–0.60, moderate; 0.61–0.80, good; and 0.81–1.00, excellent. To assess the chromosomal aberrations from the ionizing radiation, human peripheral blood cells were used in this study. In detail, phytohemagglutinin was treated for cell incubation for 44 h and treated with colcemid for an additional incubation of 4 h. After that, the cells were fixed through the air-drying method and stored in the freezer at −20 °C. For translocation assessment, fluorescence in situ hybridization was conducted on chromosomes 1, 2, and 4 and their sample images were captured. The tracking counts for the translocation and the results from the chromosomes on 1, 2, and 4 were performed according to the International System for Human Cytogenetic Nomenclature and the International Atomic Energy Agency Technical Report Series No. 405 as the analysis method [31,32]. The total amount of radiation exposure in semiconductor workers was estimated as the full genome aberration frequency, which was calculated by the translocation frequency and fraction of hybridized genome according to sex (Equation (1)). According to this equation, less than five stable chromosomes of 1000 in metaphase are below the limit of detection, and the exposure dose can be estimated as <0.1 Gy. All analyses were performed using SAS and R.

Equation (1): full genome (FG) aberration frequency according to ionizing radiation exposure:

(1) $$\mathrm{FG}=\frac{Fp}{2.05\times fp\;x\;\left(1-fp\right)}$$

where *Fp* is the translocation frequency detected by the fluorescence in situ hybridization method and *fp* is the fraction of genome hybridized according to the sex of study subjects.

## 3. Results

### 3.1. General Characteristics of Semiconductor Workers

The 306 semiconductor workers were included in this study and their median age was 36 years old, which showed significant differences between job categories (Table S4). Compared with other groups, office workers were the oldest (median age, 45 years), followed by assembly package (median age, 40 years) and non-implant processes workers (median age, 39 years). LCD workers were the youngest group (median age, 32 years) in the semiconductor industry. Among all workers, 72% consisted of males. The employment period was statistically different according to job category (*p* < 0.01) and the median employment period for all workers was 13 years. In the case of office workers, they showed the longest employment period (21 years), and LCD and non-implant processes workers showed the shortest employment period (7 years). For smoking and alcohol consumption, preventive maintenance and non-implant processes workers showed a higher prevalence rate than other groups, and the current smoking status was significant between job categories. According to the possible confounders of occupational exposure to substances, we considered various dietary factors. Only mixed grain drinks showed statistically significant between job category and office workers showed the highest proportion. There was no significant difference in the intake of meat, fish, and dairy products suspected as confounding variables in arsenic exposure. Moreover, the overall intake distribution was similar between job categories.

### 3.2. Previously Used Occupational Exposure Substances (Blood Benzene, S-Phenylmercapturic Acid, Trans, Trans-Muconic Acid, 2-Ethoxyacetic Acid, and Trichloroacetic Acid)

When we referred to the BEI standards (blood benzene, KOSHA (5 µg/L); S-phenylmercapturic acid, ACGIH (50 µg/gCr); trans, trans-muconic acid, ACGIH (500 µg/gCr)), no one was identified over the BEI level in both blood benzene and S-phenylmercapturic acid according to job category. In the case of trans, trans-muconic acid, one LCD, one non-implant processes worker, and one operator worker showed a higher BEI level, but it was not significant according to job categories. In the case of 2-ethoxyacetic acid, none of the workers were detected over the BEI level (100 mg/gCr). For TCA, which is a byproduct of TCE, just one worker who works as an engineer in non-implant processes was over the BEI level (100 mg/gCr).

### 3.3. Arsenic

Arsenic was assessed in subcontractor workers (preventive maintenance) and implant process workers as an exposed group and office and assembly package workers as an unexposed group. Except for AsB, ACGIH suggests BEI at 35 µg/L for the total arsenic, and some workers (office work: 2; assembly: 5; preventive maintenance: 24; and implant process: 6) were identified over the BEI level from the assessment (Table 1). Moreover, among workers who were identified over BEI levels in total arsenic, most of them were exposed to DMA. The composition of individuals with total arsenic levels above the BEI level were not significantly different between job categories (Table 1). In the case of inorganic arsenic (As3+ and As5+), which is suspected to have a high risk of occupational diseases, only one assembly package worker was identified over the BEI level (Table 1). The geometric mean between the detection rate over BEI and the job category was not significantly different (Table S5). Compared with the semiconductor workers, arsenic concentration of the general population and the residents around the abandoned mines seemed to be similar or higher. In a multivariable logistic regression analysis, rice, soy milk, fruit juice, and day shift work were related to total arsenic concentration.

### 3.4. Cotinine and Melatonin (6-Hydroxymelatonin Sulfate)

Cotinine level was related to job categories only in current smokers (Table 2). To define the current smoker according to cotinine level, we derived the criteria from the previous study (above 50 ng/mL of cotinine, 33). According to the definition, 52% of preventive maintenance workers, 31% of non-implant processes, and 23% of LCD workers were defined as a smoker (Table 2). Moreover, the validity of smoker definition according to cotinine assessment and self-questionnaire showed high agreement (Kappa coefficient, 0.83; 95% CI, 0.76–0.90). Due to the lack of the BEI standard, we referred to the limit of detection level from a previous study that assessed melatonin levels for 16 h from spot urine samples in normal subjects (3.42 ng/mL). The detection rate according to the job category was marginally significant after work and it showed a significant change in their status change (Table 3). Based on the reference, melatonin concentration was significantly different according to the shift work time and duration before semiconductor work only (Table 4). In addition, there was a difference in melatonin concentration according to shift work, shiftwork duration, and workers’ bedtime (Table 4). In the multivariable analysis, smoking status, weekend bedtime, vegetable intake, the first age of alcohol consumption, and shift work type were correlated with melatonin level.

### 3.5. Ionizing Radiation

**Figure 1:**
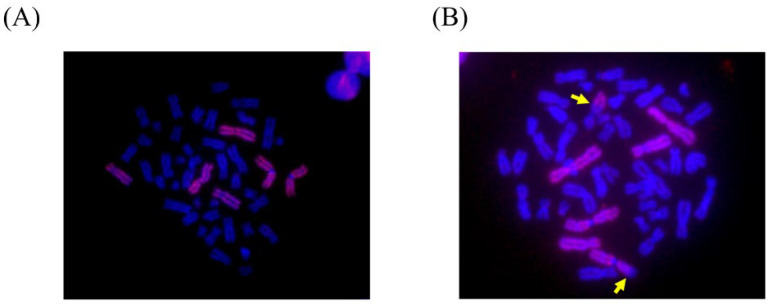
Stable chromosome translocation based on fluorescence in situ hybridization (FISH) in the microscope. (**A**), Normal; (**B**) translocation.

This study focused on the biological effects of ionizing radiation on semiconductor workers. Therefore, the number of stable chromosome translocations in metaphase cells and exposure dose were evaluated to assess the ionizing radiation exposure in semiconductor workers (Table 5). Among them, more than five stable chromosome translocations defined workers who were exposed to more than the LOD level (0.1 Gy) of ionizing radiation exposure (Figure 1). Among the 19 semiconductor workers (13 ion implants, three office workers, and three assembly package workers), only two workers were identified as having ionizing radiation exposure above the LOD level, and their estimated dose was 0.154 Gy (95% CI, 0.112–0.203). The two workers were office and ion implant workers, and both were non-smokers.

## 4. Discussion

This study aimed to assess the occupational substances in blood or urinary levels that may have been exposed to hazardous substances in semiconductor work whose employment is more than five years. As detailed occupational substances, benzene, 2-ethoxyethanol, trichloroethylene, arsenic, shift work, tobacco smoking, and ionizing radiation were selected, and the biomarkers were as follows: benzene in blood, S-phenylmercapturic acid, and the trans,trans-muconic acid in urine for benzene; trichloroacetic acid in urine for trichloroethylene; arsenic (3+) and arsenic (5+) as inorganic arsenic species, AsB, MMA, and DMA as organic arsenic species for arsenic; 6-Hydroxymelatonin sulfate in urine for shift work, cotinine for tobacco smoking, and bio-dosimetry using blood for ionizing radiation. Although some biomarkers were detected above the BEI level, there was no difference between the non-fabrication and fabrication groups and no difference across job categories. Urinary 6-hydroxymelatonin sulfate levels were much lower in workers with current shift work of swing and night and with a long employment period (≥10 years) than in workers with no shift work and with a short employment period (<5 years). Because many workers who did not work shifts were subcontractor workers, careful interpretation is needed. The cotinine levels were higher in subcontractor workers than in others, and the agreement between current smoking detection according to cotinine level and current smokers confirmed in the questionnaire was excellent (kappa index = 0.83). Moreover, it was difficult to identify specific chromosomal abnormalities due to exposure to ionizing radiation.

Among the selected substances, most were not detected over the BEI level. Trans, trans-muconic acid was identified in a few semiconductor workers. According to the individual history, there were no smokers, and they drink two or three times a month regularly. Usually, trans, trans-muconic acid is suspected of exposure related to other factors than occupational factors such as smoking, alcohol consumption, and diet [33,34,35]. In diet, trans, trans-muconic acid can be originated from the preservation of foods such as canned and sausages [35]. However, in this study, only general foods and their frequency were considered, not detailed preserved foods. In the case of TCA, one non-implant worker identified over the BEI level, and he has been drinking and smoking for more than 15 years. Considering the main sources of TCA exposure, additional assessment is mandatory based on water and grain. In the case of total arsenic, the office, assembly package (candidate unexposed group), preventive maintenance, and ion implant (candidate exposed group) groups were over the BEI level. In particular, organic arsenic was identified in an office worker who smoked for 20 years and consumed seaweed such as green laver and dried laver 2–4 times a week. When we considered speciation analysis, only one assembly package worker showed over the BEI level for inorganic arsenic. This result suggested other origins can contribute to the arsenic level. Some previous studies have suggested that smoking, soil, drinking water, and food including seafood can affect individual arsenic concentration [36,37]. Some previous studies reported that a high concentration of arsenic 5+ was identified in hijiki and gulfweed, so further consideration is needed [38,39]. However, the interpretation of arsenic, the total arsenic concentration between semiconductor workers and the general Korean population living near closed mines, the concentration level in the exposed group was similar to the general population or lower than the general population in all species of arsenic [40,41]. Most semiconductor workers were not exposed to significant ionizing radiation doses. Although two workers were exposed to the LOD level, their exposure dose was close to the LOD level. Therefore, we cannot assess the significant ionizing radiation in semiconductor workers. When comparing retired European nuclear power plant workers and smokers in the general population, there was no significant difference in their chromosomal aberrations (mean, 2.32; standard deviation, 1.86). As the semiconductor industry has developed, occupational diseases have continuously raised. However, since occupational exposure substance is difficult to identify in the semiconductor manufacturing process, it is limited to assess the causality between occupational substances and occupational disease [42]. The main causes of the difficulties were the change in the semiconductor manufacturing process, patents, and corporate secrets [42]. Thus, to select candidate occupational substances we referred to the carcinogen and reproductive toxicity classification from IARC, KOSHA, and occupational environment experts’ opinions. Previous studies have suggested ionizing radiation, benzene, butadiene, dichloroethane, and formaldehyde as occupational exposure materials [43]. However, it is difficult to evaluate in the semiconductor facilities because the actual exposure level can be similar to or lower than the general environment. Moreover, these materials can be originated from other environmental sources such as food, water, and airborne substances [44]. In a previous study, heavy metals such as gallium, indium, and arsenic were identified in urine samples from Taiwanese semiconductor workers [45]. Additionally, other factors such as melatonin or cotinine which are known as non-hazard materials, suggest as an additional biomarker to evaluate semiconductor workers’ health effects such as shift work or smoking status.

In addition, most of the previous epidemiological studies of semiconductor workers have been based on the general population, which presented a standardized incidence ratio or mortality ratio [7,8,46,47]. Therefore, they did not consider an appropriate comparison group. To assess the detailed exposure information, consideration of various work processes and job categories is mandatory [48,49]. The semiconductor industry consists of various processes and the solvents used are different according to each process. Therefore, it is important to select the study population which considered various conditions to assess the occupational disease. Many previous semiconductor studies have not considered this, for example, some studies defined overall semiconductor as fab work or a few job categories only [8,47,49]. However, this definition overlooks the detailed exposure according to each process in fab work. Moreover, in most studies, non-fab workers are defined as office workers only [50,51]. In addition, some studies have suggested the standardization ratio based on the general population, which was inappropriate as a comparison group from semiconductor workers [7,8,50]. In this study, office and assembly package work is defined as non-fab work (unexposed group), and fab work was classified into various processes.

Nevertheless, there are several limitations in this study, first, we could not consider additional occupational substances. Because of realistic measurement, the half-life of occupational substance should be considered. Second, it was difficult to select more samples for the study population due to the semiconductor working environment, which works 24 h in three shifts. It was particularly difficult to select the study population similarly in each stratum such as sex, job category, and shift work type. Since chromosome translocation should be assessed directly with the eyes, the small size of translocation could be overlooked; therefore, there must be awareness for interpretation. Although we could not find significant occupational exposure in semiconductor workers, additional causal assessment is needed regarding smoking, drinking, and eating habits. However, this study evaluated various occupational substances in semiconductor workers based on the BEI. Those substances are described as carcinogens and reproductive toxicity by IARC and KOSHA, so it has objectivity and validity. In addition, it can represent overall semiconductor workers because the study population was considered for various job categories compared with previous studies. Moreover, this study suggests the significance of melatonin and cotinine in further study to consider workers’ working environment.

## 5. Conclusions

In this study, occupational substances were assessed in current semiconductor workers based on the BEI level, and just a few workers were detected over the BEI level. However, exposure to other sources is suspected rather than occupational environmental exposure. Just one assembly package worker was identified as inorganic arsenic. In the case of radiation exposure, there was no significant difference in their chromosome aberrations compared with the European nuclear power plant retired workers and smokers in the general population. Moreover, melatonin concentration was related to the shift work, and cotinine could be a surrogate marker of smoking status evaluation. To assess the association between occupational substances and occupational diseases in semiconductor workers, a comprehensive selection strategy that represented the overall semiconductor process is needed. Moreover, additional re-measurement is considerable to evaluate other sources of each occupational substance in workers who identified above the BEI level. Next, a prospective cohort design that considers these conditions and additional material assessment is necessary. Consequently, this consideration will be able to evaluate the detailed health effects of semiconductor workers.

## Figures and Tables

**Table 1:** Detection rate and the status change in the urinary total arsenic concentration above the biological exposure index (BEI) according to job category.

	Detection Rate over BEI		Status Change in Arsenic Concentration (below/over BEI) before and after Work			
	Before Work	After Work	Below-Below	Below-Over	Over-Below	Over-Over
	N (%)	N (%)	N (%)	N (%)	N (%)	N (%)
Total arsenic concentration ^1^ (AsB not included)						
Office work (*n* = 12)	2 (17)	-	-	-	-	-
Assembly package (*n* = 12)	3 (25)	4 (33)	7 (58)	2 (17)	1 (8)	2 (17)
Preventive maintenance (*n* = 67)	22 (33)	3 (4)	43 (64)	2 (3)	21 (31)	1 (1)
Implant process (*n* = 13)	6 (46)	3 (23)	7 (54)	0 (0)	3 (23)	3 (23)
*p*-value ^3^	0.16	0.7	0.92			
Total inorganic arsenic ^2^						
Office work (*n* = 12)	0 (0)	-	-	-	-	-
Assembly package (*n* = 12)	0 (0)	1 (8)	11 (92)	1 (8)	0 (0)	0 (0)
Preventive maintenance (*n* = 67)	0 (0)	0 (0)	67 (100)	0 (0)	0 (0)	0 (0)
Implant process (*n* = 13)	0 (0)	0 (0)	13 (100)	0 (0)	0 (0)	0 (0)
*p*-value ^3^	NA	NA	NA			

Abbreviations: BEI: biological exposure index; AsB: arsenobetaine; NA: not applicable. ^1^ Total arsenic concentration consisted of arsenic (As3+ and As5+), BEI defined by the American Conference of Governmental Industrial Hygienists criterion (using Threshold Limit Values) = 35 μg/L. ^2^ Inorganic arsenic concentration consisted of arsenic (As3+ and As5+) and the BEI defined by the Agency for Toxic Substances and Disease Registry, US criterion = 10 μg/L. ^3^ *p*-value defined the arsenic concentration difference according to the job categories.

**Table 2:** Geometric mean and detection rate of urinary cotinine in current smokers among semiconductor workers.

	Non-Smokers	Former Smokers	Current Smokers	Current Smokers ^1^	
				Cotinine ^1,2^	Questionnaire ^2^
	GM (P25–P75)	GM (P25–P75)	GM (P25–P75)	N (%)	N (%)
Office work (*n* = 24)	1 (ND-1)	4 (1–12)	136 (136–136)	2 (8.3)	1 (4.2)
Assembly package (*n* = 43)	1 (ND-ND)	5 (ND–71)	152 (151–152)	5 (11.6)	3 (7.0)
Preventive maintenance (*n* = 67)	1 (ND-ND)	2 (ND–ND)	138 (132–150)	35 (52.2)	37 (55.2)
Implant process (*n* = 22)	1 (ND-ND)	2 (ND–2)	111 (104–121)	3 (13.6)	2 (9.1)
Non-implant processes (*n* = 58)	1 (ND-1)	12 (1–94)	112 (101–129)	18 (31.0)	15 (25.9)
LCD (*n* = 43)	1 (ND-1)	12 (ND–124)	131 (127–133)	10 (23.3)	6 (14.0)
Operator (*n* = 45)	1 (ND-1)	9 (22–66)	131 (127–133)	1 (2.2)	0 (0)
Total (*n* = 302)	1 (ND-1)	5 (ND–89)	131 (121–148)	74 (24.5)	64 (21.1)
*p*-value	0.74	0.15	0.006		

Abbreviations: GM: geometric mean; ND: not detectable. ^1^ The current smoker is defined by the cotinine level in urine (≥50 ng/mL). ^2^ The kappa statistic (0.83 (95% CI, 0.76–0.90)) in the current smokers calculated between smokers identified in cotinine level and the self-reported questionnaire.

**Table 3:** Detection rate and the status change in the urinary 6-hydroxymelatonin sulfate levels under the reference according to job category.

	Detection Rate under the Reference ^1^		Status Change in 6-Hydroxymelatonin Sulfate (under the Reference ^1^) before and after Work		
	Before Work	After Work	Normal-Normal	Normal-Detection	Detection-Normal
	N (%)	N (%)	N (%)	N (%)	N (%)
Office work (*n* = 24)	1 (4)	-			
Assembly package (*n* = 43)	1 (4)	0 (0)	42 (96)	0 (0)	1 (4)
Preventive maintenance (*n* = 67)	0 (0)	0 (0)	67 (100)	0 (0)	0 (0)
Implant process (*n* = 22)	1 (5)	2 (7)	20 (88)	2 (7)	1 (5)
Non-implant processes (*n* = 57)	3 (5)	0 (0)	54 (95)	0 (0)	3 (5)
LCD (*n* = 43)	2 (4)	1 (2)	41 (95)	1 (2)	2 (4)
Operator (*n* = 46)	3 (5)	0 (0)	43 (95)	0 (0)	3 (5)
*p*-value ^2^	0.20	0.04	0.02		

^1^ The reference was 3.42 ng/mL based on general population (average urine volume of 300 cc per four hours). ^2^ *p*-value defined as the difference of 6-hydroxymelatonin sulfate levels among job categories in each work.

**Table 4:** The detection rate of the urinary 6-hydroxymelatonin sulfate levels for semiconductor workers according to the shiftwork and the bedtime.

	N	GM (P25–P75)	*p*	Low 1	Normal	*p*
				N (%)	N (%)	
Shiftwork at the Survey Date						
No shiftwork ^2^	76	200 (45–1102)				
Day	69	47 (22–111)	0.01	2 (2.9)	67 (97.1)	0.03
Swing	71	19 (8–33)		3 (4.2)	68 (95.8)	
Graveyard	69	24 (6–58)		5 (7.2)	62 (92.5)	
Shiftwork duration (years) except PM ^3^						
<5	12	21 (8–40)	0.02	0 (0)	12 (100)	0.04
5–9	82	19.5 (7–41)		4 (4.9)	78 (95.1)	
≥10	115	15.5 (5–43)		8 (7.0)	107 (93.0)	
Bedtime last weekend except for PM						
Before midnight	130	46 (9–136)	0.02	-		
After midnight	79	42 (9–150)		-		

Abbreviations: GM: geometric mean; PM: preventive maintenance. ^1^ Lower than the reference (3.42 ng/mL) defined as the ‘Low’ group. ^2^ Office workers, manufacturing workers who do not work shifts, and PM workers who do not work shifts. ^3^ Only current shiftwork workers were assessed.

**Table 5:** Chromosome translocation and estimated exposed dose according to the radiation exposure in semiconductor workers.

ID ^1^	Age	No. of Metaphase Cell	No. of Stable Chromosome Translocation	No. of Stable Chromosome Translocation Cell	Frequency of Stable Chromosome Aberration (per 1000 Cells)	Estimated Radiation Dose (Gy) ^2^	Smoking Status
1	41	1008	6	6	0.006	0.154 (0.112–0.203)	Non-smoker
2	39	1002	2	2	0.002	<0.1	Non-smoker
3	39	1002	2	2	0.002	<0.1	Non-smoker
4	34	1014	0	0	0	<0.1	Smoker ^3^
5	30	1007	2	2	0.002	<0.1	Non-smoker
6	36	1003	0	0	0	<0.1	Non-smoker
7	50	1010	2	2	0.002	<0.1	Non-smoker
8	42	978	1	1	0.001	<0.1	Non-smoker
9	38	1006	3	3	0.003	<0.1	Non-smoker
10	38	1061	1	1	0.0009	<0.1	Non-smoker
11	37	1009	4	4	0.004	<0.1	Non-smoker
12	34	1008	0	0	0	<0.1	Smoker ^3^
13	33	992	3	2	0.003	<0.1	Non-smoker
14	37	1009	1	1	0.001	<0.1	Non-smoker
15	35	1007	1	1	0.001	<0.1	Non-smoker
16	47	1009	6	5	0.006	0.154 (0.112–0.203)	Non-smoker
17	46	1005	1	1	0.001	<0.1	Smoker ^3^
18	40	1007	4	4	0.004	<0.1	Non-smoker
19	41	1005	4	4	0.004	<0.1	Non-smoker
Mean (SD)		1007 (15)	2.26 (1.85)	2.16 (1.85) ^4^	0.0022 (0.0018)		

^1^ All workers were male workers. ^2^ Estimated radiation dose < 0.1 Gy was defined as less than five stable chromosome translocation cells of 1000 metaphase cell. ^3^ Number of cigarettes per day (sequential, 10 cigarettes; 2 cigarettes; 15 cigarettes). ^4^ Number of stable chromosome translocation cells from a previous study of European nuclear power plant workers and smokers in the general population (Mean (SD), 2.32 ± 1.86).

## Data Availability

No new data were created or analyzed in this study. Data sharing is not applicable to this article.

## Supplementary Materials

The following supporting information can be downloaded at: https://www.mdpi.com/article/10.3390/ijerph19148737/s1, Table S1: The characteristics of selected chemical material from semiconductor workers; Table S2: Study population of selected chemical material in semiconductor industry; Table S3: Number of sample collection according to job category and collection time; Table S4: General characteristics of South Korean semiconductor workers (SC) according to their job category; Table S5: Arsenic concentration according to job category in semiconductor workers and general population; Table S6: Brief analysis tool, preprocessing, and analysis method in each occupational substance in the study; Figure S1: hierarchy of selection of semiconductor workers; Figure S2: Usual semiconductor’s shift work pattern and considered sample collection protocol in semiconductor workers.
