# Supplementary material for: Biological Assessment of Potential Exposure to Occupational Substances in Current Semiconductor Workers with at Least 5 Years of Employment
Source: Int J Environ Res Public Health. 2022 Jul 18;19(14):8737. doi: 10.3390/ijerph19148737 (PMC9318163; doi:10.3390/ijerph19148737)
Supplement: Supplementary file 1 [file ijerph-19-08737-s001.zip › ijerph-1789280-supplementary.pdf]

Table S1. The characteristics of selected chemical material from semiconductor workers.

Target material (Chemical formula)	CAS No	Process in semiconductor	Related disease	Carcinogen	Reproductive toxicity	Selected biomarker (Byproduct)	LOD	BEI
Trichloroethylene (CCl_2CHCl)	79-01-6	Mold, Chip assembly	Liver, kidney, lung, lymphoma	1B	1B	Trichloroacetic acid	1.02 μg/L	15 mg/L[1]
2-ethoxyethanol ($C_4H_{10}O_2$)	110-80-5	Photolithography	Hematology, reproductive toxicity, kidney, liver	-	1B	2-ethoxyacetic acid	0.10 mg/L	100 mg/gCr [1,2]
Benzene (C_6H_6)	71-43-2	Photolithography	Leukemia	1A	2	S-phenylmercapturic acid<br>Trans, trans-muconic acid<br>Blood benzene	0.16 μg/L<br>1.28 μg/L<br>0.33 μg/L	25 μg/gCr [1]<br>500 μg/gCr [1]<br>5 μg/L [2]
Arsenic (As)	7440-38-2	Ion implant	Lung	1A	-	Arsenic (3+)<br>Arsenic (5+)<br>Monomethylarsonic acid<br>Dimethylarsine acid<br>Arsenobetaine	0.15 μg/L<br>0.11 μg/L<br>0.16 μg/L<br>0.25 μg/L<br>0.67 μg/L	35 μg/L [1]
Ionizing radiation	-	Ion implant	All type of cancer	1A	-	Chromosome translocation from the metaphase cell	0.1 Gy	
Smoking status	-	Smoking	Lung	1	-	Cotinine	0.1 ng/mL	
Melatonin	-	Shift work	Breast	-	-	6-Hydroxymelatonin sulfate	3.42 ng/mL	

Abbreviation, LOD, Limit of detection; BEI, Biological exposure index

1. BEI is derived from American Conference of Governmental Industrial Hygienists
2. BEI is derived from Korea Occupational Safety and Health Agency

Table S2. Study population of selected chemical material in semiconductor industry.

Target material	Selected biomarker	No of study population	Targeted study population
**Blood sample**			
Ionizing radiation [1]	Chromosome translocation	13	Exposed group: Ion implant for facility worker and subcontractor workers
		6	Unexposed group: Office and assembly workers
Benzene [2]	Blood benzene	237	All workers except subcontractor workers
**Urine sample**			
Benzene [2]	Trans, trans-muconic acid	304	All workers
	S-phenylmercaptric acid	304	All workers
Trichloroethylene [2]	Trichloroacetic acid	304	All workers
2-ethoxyethanol [2]	2-ethoxyacetic acid	304	All workers
Arsenic [2]	Inorganic and organic arsenic	80	Exposed group: Ion implant for facility worker and subcontractor workers
		24	Unexposed group: Office [3] and assembly workers
Melatonin	6-Hydroxymelatonin sulfate	302	All workers
Smoking status [3]	Cotinine	302	All workers

1. Bio-specimen was collected after work time as whole-blood
2. Bio-specimen was collected before and after work time
3. Bio-specimen was collected before work time

Table S3. Number of sample collection according to job category and collection time.

Job category	Collection time	No of workers	No of SST tube (10 cc)	No of EDTA tube (10 cc)	No of EDTA tube (5 cc)	No of Conical tube (50 cc)	No of Heparin tube (10 cc)
Office	Before work	24	2	1	1	1	1 [1]
Production	Before work	215	2	1	1	1	
Production	After work	215	2	1	1	1	1 [1]
Subcontractor	Before work	67				1	
Subcontractor	After work	67				1	

Abbreviation, SST, Serum separate; EDTA, Ethylenediaminetetraacetic acid

1. Heparin tube was collected in office, assembly workers (unexposed group) and ion implant workers (exposed group) for bio-dosimetry to assess ionizing radiation

Table S4. General characteristics of South Korean semiconductor workers (SC) according to their job category.

	Overall (N=306)	Office work (N=24)	Assembly package (N=43)	Operator (N=47)	LCD (N=44)	Non-implant processes (N=59)	Implant process (N=22)	Preventive maintenance (N=67)	
	**Median**	**Median**	**Median**	**Median**	**Median**	**Median**	**Median**	**Median**	***p***
Age (years)	36	45	40	37	32	39	35	35	<0.01
BMI (kg/m²)	24	23	24	22	24	24	23	24	0.16
Employment (years)	13	21	20	17	7	7	12	11	<0.01
Working hours/week	48	45	48	48	48	48	48	48	0.19
	**%**	**%**	**%**	**%**	**%**	**%**	**%**	**%**	
Experience in shifting work	80	4	100	100	100	98	100	43	<0.01
FAB work (Over 80% in work time)	71	NA [1]	26	100	100	100	100	91	0.13
Women	28	50	49	100	0	0	0	0	<0.01
Ever smokers	42	33	21	4	27	58	50	79	<0.01
Smoking duration in men (years)									<0.01
< 10	24	25	13	NA	25	18	55	23	
10-19	63	13	50	NA	75	82	36	64	
≥ 20	13	62	37	NA			9	13	
Current drinkers	84	92	70	83	77	88	77	91	0.11
Drinking duration (years)									<0.01
< 15	36	9	13	18	71	44	53	38	
≥ 15	64	91	87	82	29	56	47	62	
Dietary intake (at least 1 time per week)									
Milk	46	42	44	34	61	48	32	52	0.07
Liquid yogurt	22	25	14	21	27	19	14	27	0.63
Semisolid yogurt	13	17	14	11	11	10	18	13	0.72
Soy product	56	75	56	53	43	56	68	57	0.35
Fish	31	38	42	28	20	36	23	30	0.35
Meat	59	63	51	66	52	66	41	63	0.15
Mixed grain drink	64	88	56	74	66	67	68	48	0.01

All kind of vegetables	84	92	88	74	75	92	73	87	0.06

Abbreviation: FAB, Fabrication; NA, Not applicable

1. Office workers was not applicable for the most of working environment questionnaire

Table S5. Arsenic concentration according to job category in semiconductor workers and general population.

	**Semiconductor work according to the job category [1]**				**Population other than semiconductor workers**	
	Office work	Assembly package	Preventive maintenance	Implant process	General population [2]	Population near abandoned metal mine [3]
	(N=12)	(N=12)	(N=67)	(N=13)	(N=2,077)	(N=974)
	**GM (P25-P75)**	**GM (P25-P75)**	**GM (P25-P75)**	**GM (P25-P75)**	**GM (GSD)**	**GM (P25-P75)**
**Inorganic arsenic (3+)**	0.82 (0.49-0.89)	1.42 (0.90-2.15)	1.06 (0.63-1.55)	1.21 (0.77-1.75)	1.04 (9.63)	0 (0-0.01)
**Inorganic arsenic (5+)**	0.73 (0.10-1.98)	0.49 (0.26-0.8)	0.6 (0.32-1.06)	0.71 (0.55-0.90)	0.72 (5.40)	2.66 (1.82-3.87)
**Organic arsenic (MMA)**	1.97 (0.98-2.83)	2.74 (1.72-4.69)	2.14 (1.05-3.5)	2.94 (2.13-6.08)	1.46 (4.00)	2.02 (1.60-2.55)
**Organic arsenic (DMA)**	15.45 (11.39-18.51)	24.61 (21.45-28.16)	18.77 (12.21-36.17)	24.04 (12.26-32.43)	26.64 (2.68)	36.37 (31.99-41.36)
**Organic arsenic (AsB)**	51.23 (29.38-145.55)	30.56 (18.82-46.06)	30.55 (13.44-57.52)	43.59 (25.42-47.53)	45.34 (4.36)	-

Abbreviation, GM, Geometric mean; GSD, Geometric standard deviation; MMA, monomethylarsonic acid; DMA, dimethylarsinic acid; AsB, Arsenobetaine

1. *P-value* of the arsenic concentration difference according to the job categories was not significant
2. The Korean population which selected stratified random sampling defined as the general population (Men are 44% in the population and the average age was 45.5 years)
3. The population near abandoned mine derived from the environmental and health effects survey between 2013 and 2014 (Men are 41% in the population and the average age was 67.4 years)

Table S6. Brief analysis tool, preprocessing, and analysis method in each occupational substance in the study.

Target material	Preprocessing method	Analysis	Analysis tool
Blood benzene	Headspace	After preprocessing of the sample, purified water, NaCl, K_2CO_3 were mixed and analyzed by Headspace- GC-MS	Perkin Elmer HS GC-MS SQ8T
S-phenylmercapturic, Trans, trans-muconic acid	Solid Phase extraction	After preprocessing of the sample, methanol and 0.1% acetic acid were treated first then 10% acetic acid was used for elution as an analysis specimen. All of the analysis was based on the HPLC-MS	Agilent LC-MS/MS 6410
Trichloroacetic acid	liquid-liquid extraction	After preprocessing, n-hexane was treated and centrifuged at 2,000 rpm for analysis. All of the analysis was based on the GC-ECD	Agilent GC-ECD 5890
2-ethoxyacetic acid	liquid-liquid extraction	After preprocessing, methylene chloride and isopropyl alcohol were treated and centrifuged at 3,000 rpm for analysis. All of the analyses based on the GC-MS	Perkin Elmer GC-MS SQ8T
Arsenic	Dilution	Arsenic speciation analysis based on the LC-ICP/MS	Perkin Elmer ICP-MS NexoION 2000
Melatonin	-	Dilute all samples at 1:200 with incubation buffer. Remove excess strips from the holder and reseal them in the foil pouch together. Use refrigerated reagent solutions. Add antiserum to all wells except blank wells. Allow the TMB substrate solution to reach 18-28 degrees Add stop solution to all wells. Remove air bubbles with a pipette tip.<br>Read the absorbance at 450 nm in a microtiter plate reader. All analysis was conducted in ELISA	BUHLMANN ELISA EK-M6S
Cotinine	-	Centrifuging in 2,500-3,000 rpm for 20 minutes. All analysis was conducted in ELISA	Absorbance ELISA microplate reader
Creatinine	-	Modified Jaffe method	Roche Cobas 702

Na, K, Cl	Ion-selective electrodes	Roche Cobas 702

Abbreviation, Headspace-GC/MS, Headspace-Gas Chromatography/Mass Spectrometer; HPLC-MS, High-Performance Liquid Chromatography-tandem Mass Spectrometer; GC-ECD, Gas Chromatography/Electron Capture Detector; LC-ICP/MS, Liquid Chromatography-Inductively Coupled Plasma/Mass Spectrometer; ELISA, Enzyme-linked immunosorbent assay

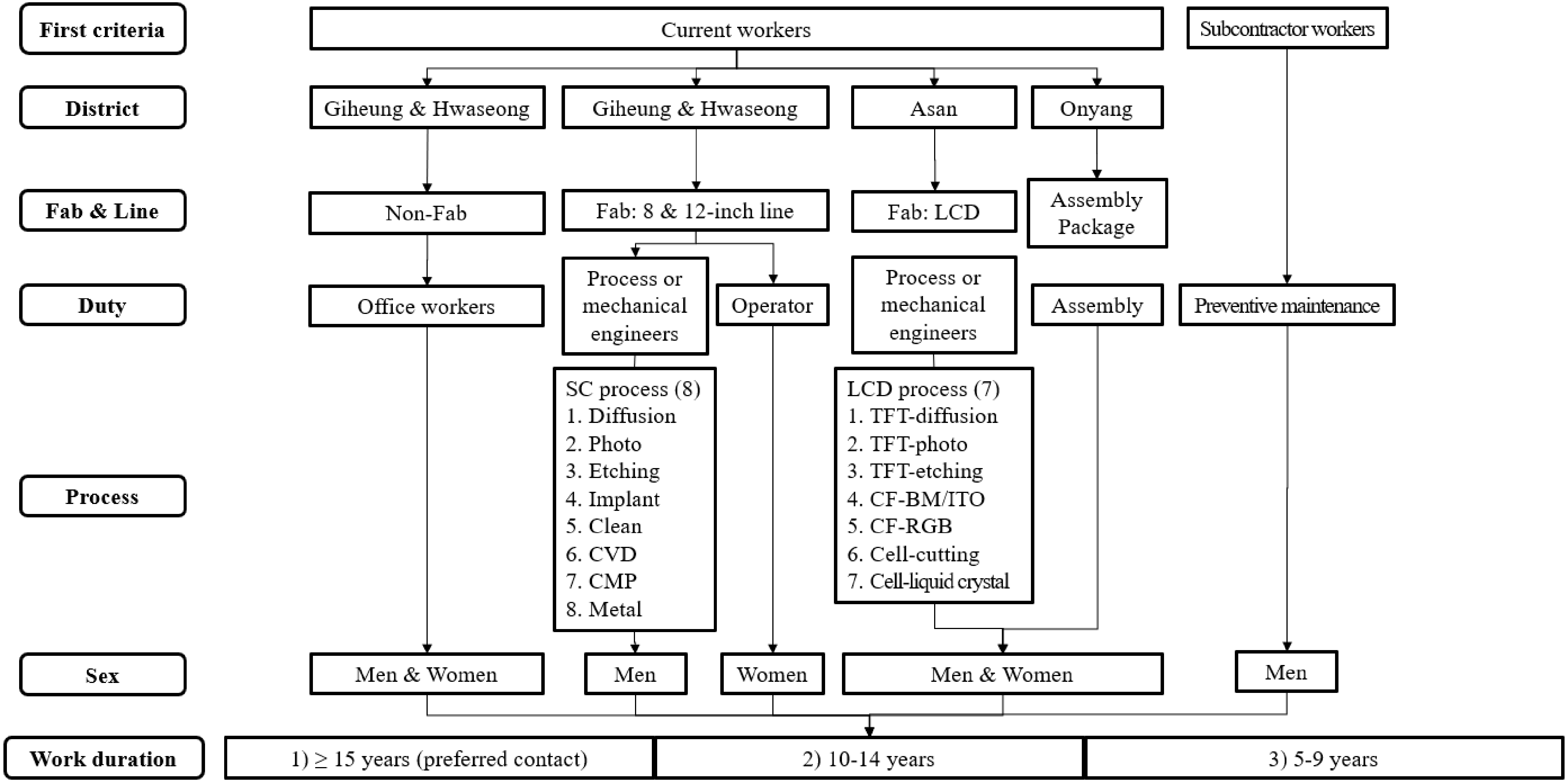

Figure S1. Hierarchy of selection of semiconductor workers.

Semiconductor work pattern (example)

Group	Day 1	Day 2	Day 3	Day 4	Day 5	Day 6	Day 7	Day 8
A	Swing	Swing	Off	Off	Night	Night	Night	Night
B	Night	Night	Night	Night	Off	Off	Day	Day
C	Day	Day	Day	Day	Day	Day	Off	Off
D	Off	Off	Swing	Swing	Swing	Swing	Swing	Swing

Sample collection protocol in the study considering semiconductor shift work

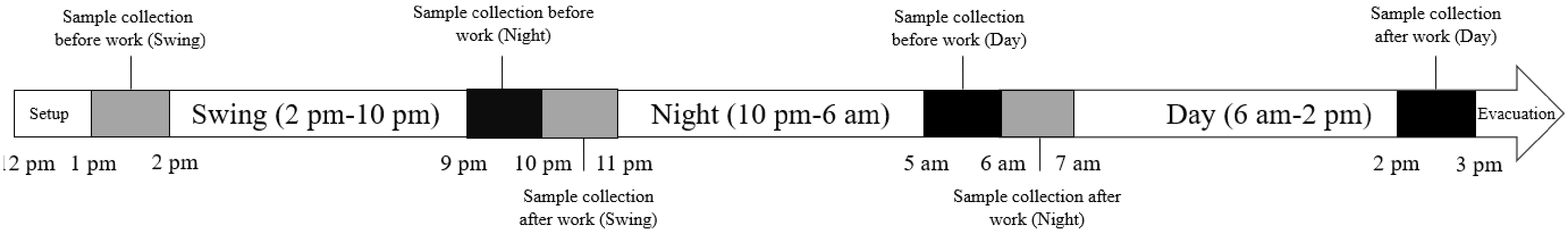

Figure S2. Usual semiconductor's shift work pattern and considered sample collection protocol in semiconductor workers.
